# Analysis of audiological outcomes of children referred from a universal newborn hearing screening program over 9 years in Beijing, China

**DOI:** 10.1038/s41598-023-50171-8

**Published:** 2023-12-19

**Authors:** Yue Li, Xiaozhe Yang, Chuan Wang, Xiaohua Cheng, Beier Qi, Hui En, Cheng Wen, Yiding Yu, Lin Deng, Dongxin Liu, Xinxing Fu, Hui Liu, Lihui Huang

**Affiliations:** 1 Department of Otolaryngology-Head and Neck Surgery, Beijing Tongren Hospital, Capital Medical University, Beijing, China; 2 Beijing Institute of Otolaryngology, Beijing, China; 3 Key Laboratory of Otolaryngology Head and Neck Surgery, Ministry of Education, Beijing, China; 4 Maternal and Child Health Hospital of Chao Yang District, Beijing, China; 5 Ear Science Institute Australia, Subiaco, WA Australia

**Keywords:** Diagnosis, Paediatrics, Auditory system, Risk factors

## Abstract

Universal newborn hearing screening (UNHS) and audiological diagnosis are crucial for children with congenital hearing loss (HL). The objective of this study was to analyze hearing screening techniques, audiological outcomes and risk factors among children referred from a UNHS program in Beijing. A retrospective analysis was performed in children who were referred to our hospital after failing UNHS during a 9-year period. A series of audiological diagnostic tests were administered to each case, to confirm and determine the type and degree of HL. Risk factors for HL were collected. Of 1839 cases, 53.0% were referred after only transient evoked otoacoustic emission (TEOAE) testing, 46.1% were screened by a combination of TEOAE and automatic auditory brainstem response (AABR) testing, and 1.0% were referred after only AABR testing. HL was confirmed in 55.7% of cases. Ears with screening results that led to referral experienced a more severe degree of HL than those with results that passed. Risk factors for HL were identified in 113 (6.1%) cases. The main risk factors included craniofacial anomalies (2.7%), length of stay in the neonatal intensive care unit longer than 5 days (2.4%) and birth weight less than 1500 g (0.8%). The statistical data showed that age (*P* < 0.001) and risk factors, including craniofacial anomalies (*P* < 0.001) and low birth weight (*P* = 0.048), were associated with the presence of HL. This study suggested that hearing screening plays an important role in the early detection of HL and that children with risk factors should be closely monitored.

## Introduction

Hearing loss (HL) is the most common sensory defect worldwide. The World Report on Hearing indicates that the global prevalence of moderate or high-grade HL increases with age among children, rising from 0.2% at birth to 1.5% at age of 5 years^1^. The WHO noted that 34 million children are living with disabling HL^1^. Children with undiagnosed HL are likely to have speech development delays, which may affect their physical health, emotional development and quality of life^2–4^. Universal newborn hearing screening (UNHS) programs have been implemented in China and many other countries worldwide and have contributed substantially to the early detection and diagnosis of congenital HL^5,6^. Studies from high-income nations such as Australia, the Netherlands, the United Kingdom, and the USA, as well as middle-income nations such as India, Nigeria, and the Philippines, have demonstrated how cost-effective newborn hearing screening is^1,7^. For instance, long-term cost–benefit studies of UNHS programs in China have also been reported^8–10^.

In China, UNHS programs have been implemented in the past 20 years and have brought about clear benefits in terms of early diagnosis among children with congenital HL and a reduced burden on society^11–13^. The ‘Technical Specifications for Newborn Hearing Screening’ were first formulated by the former Ministry of Health of China in 2004, and subsequently, the ‘Technical Specifications for Newborn Hearing Screening (2010 Edition)’ were issued in 2010^10^. In 2012, concurrent hearing and genetic screening in the whole newborn population in Beijing was initiated, and subsequently, other cities in China also implemented this screening^14–17^. In 2013, the former National Health and Family Planning Commission promulgated the Technical Standards for Children's Ear and Hearing Health Care, incorporating the ear and hearing care of children aged 0–6 years into the health care system^18^. After years of promotion, UNHS programs in China were generally reported to be of fair quality, consistent with international UNHS programs, although there needs to be further improvement in terms of ‘stakeholder involvement, developmental rigor, and editorial independence’^19^.

In the UNHS program in Beijing (China), normal newborns who were born healthy without risk factors for HL, should be screened in two stages: the initial screening should be completed from 48 h after birth to before discharge; those who fail the screening and those who miss the screening should be rescreened within 42 days^11^. Since the timing of maternal postpartum reexamination and the growth and developmental health check-up of newborns at age of 42 days, hearing rescreening by the age of 42 days would facilitate rescreening rates^11,19,20^. The predominantly used screening techniques in the UNHS program in Beijing (China) are either transient evoked otoacoustic emission (TEOAE) or automatic auditory brainstem response (AABR) testing or a combination of TEOAE and AABR testing, which was in accordance with ‘Technical Specifications for Newborn Hearing Screening (2010 Edition)’. Infants who do not pass the two-step hearing screening undergo diagnostic audiological tests by the age of 3 months in tertiary referral centers. Newborns in the neonatal intensive care unit (NICU) should be screened by AABR before discharge, if the condition permits, a combination of TEOAE and AABR testing would be used. Briefly, if the newborns in the NICU did not pass the hearing screening, they were directly referred to tertiary referral centers for hearing diagnosis^11^. The Pediatric Audiology Clinic of the Ear, Nose, and Throat (ENT) Department in our hospital (Beijing Tongren Hospital, Capital Medical University) is a tertiary referral center in Beijing. Auditory brainstem response (ABR), auditory steady-state response (ASSR), distortion product otoacoustic emission (DPOAE), and acoustic immittance testing are performed to determine the presence, laterality, type and degree of HL, and cochlear microphonic (CM) is performed in view of auditory neuropathy spectrum disorder (ANSD) diagnosis.

This research examined the hearing screening techniques and audiological test results among children who were referred to our hospital after failing UNHS over a 9-year period (2013–2021) to better understand the audiological characteristics and identify relevant risk factors for HL in cases referred from the UNHS program to potentially raise awareness in the practice of children’s ear and hearing care.

## Materials and methods

### Study population

In this study, we included 1839 cases who were referred to Beijing Tongren Hospital, Capital Medical University (Beijing, China) after failing UNHS from January 2013 to December 2021. The cases underwent audiological assessment at the Clinical Audiology Center and were subsequently diagnosed by experienced ENT doctors in the Pediatric Audiology Clinic. Information on the hearing screening techniques, screening results in UNHS and audiological results of these cases was collected. In addition, we collected demographic information from the cases, including age (months), date of birth, date of first hospital visit, sex, risk factors for HL and temporal bone computed tomography (CT) or inner ear magnetic resonance imaging (MRI) scan results. The study was approved by the Ethics Committee of Beijing Tongren Hospital, Capital Medical University. This study was performed in accordance with the Declaration of Helsinki.

### UNHS

UNHS was performed using three different hearing screening techniques: TEOAE, AABR or a combination of TEOAE and AABR testing. The relevant testing parameters of TEOAEs were as follows: a ‘click’ was used for acoustic stimulation with a stimulus intensity of a 70–75-decibel (dB) sound pressure level (SPL) and background noise < 40 dB (A); the passing criteria were total reaction intensity ≥ a 10-dB SPL, repetition rate ≥ 50%, and signal-to-noise ratio (SNR) (at least 3 frequencies) ≥ 3 dB^16,21,22^. The relevant testing parameters of AABR were as follows: a ‘click’ was used for acoustic stimulation with a stimulus intensity of a 35-dB normalized hearing level (nHL) and stimulation rate of 93 times/s; background noise < 45 dB (A), sampling rate of 16 kHz and a spectrum ranging from 700–750 Hz to 5000 Hz^16,23^.

### Preassessment procedures

Concerning the procedures, the basic intake information of all cases was recorded. Data on risk factors for HL were collected according to the criteria recommended by the ‘Technical Specifications for Newborn Hearing Screening (2010 Edition)’: craniofacial anomalies, including auricle and canal anomalies; length of stay in the NICU longer than 5 days; birth weight less than 1500 g; mechanical ventilation time longer than 48 h; HL-associated syndromes in the clinic; hyperbilirubinemia that met requirements for blood exchange; bacterial meningitis; and in utero infection with cytomegalovirus and toxoplasmosis^11^. Subsequently, otoscopic examinations were performed to identify common problems (e.g., perforations, impacted earwax) of the ear canal and middle ear. Informed consent was obtained from parents/legal guardians for all cases included in this study.

### Audiological assessments

In this study, cases underwent a series of diagnostic audiological assessments to determine the presence, laterality, type and degree of HL, and assessment results including click-evoked ABR (c-ABR), DPOAEs, and acoustic immittance from the first diagnostic audiological assessment for each child were included as data in this study. c-ABR and DPOAE assessments were carried out in acoustically treated test rooms with ambient noise levels below 30 dB (A), while acoustic immittance assessments were carried out in a quiet room.

c-ABR testing was carried out while the cases were asleep. Chloral hydrate (50–100 mg/kg of weight) was given sometimes. The procedure consisted of recording a series of waves representing the auditory pathway using surface electrodes located at the vertex and on the ipsilateral mastoid. The test lasted approximately 30–45 min. The stimulus used was a short alternating click transmitted to the newborn via headphones placed in each ear, masking the unexplored contralateral ear with white noise. The test was initiated by applying the stimulus at intensities of 90 dB nHL, decreasing the intensity progressively and identifying the presence of wave V, which disappears progressively as the intensity of the stimulus decreases, and considering the auditory threshold for the explored ear when it was still possible to identify wave V at low-stimulus intensities. When this hearing threshold exceeded 30 dB nHL, it was considered abnormal^24,25^. c-ABR testing was performed using evoked potential tests (Nicolet Spirit, Nicolet Inc., Madison, WI, USA), and the assessment results were recorded on the audiogram in numerical format to facilitate data entry. The 100-ms clicks were presented monaurally at a repetition rate of 19.3/s via insert earphones. The stimulus was calibrated by measuring the peak equivalent SPL. The 0-dB nHL of this stimulus was set up by a group of young adults with normal hearing and had a peak equivalent SPL of 32 dB. Therefore, the maximum output level for the clicks was an SPL of 132 dB (peak). Two runs of alternating polarity testing were conducted at each presentation level for waveform identification. The ABR threshold was identified as the lowest level at which wave V could be detected visually. The step-in presentation level was 10 dB. An absent response was defined as no repeatable waveform for at least two runs at the maximum presentation level. Click-evoked ABR testing using both condensation and rarefaction single-polarity stimuli to determine whether CM presents in view of auditory neuropathy spectrum disorder (ANSD) diagnosis. The hallmark of the ANSD ABR is a prominent CM that follows the stimulus polarity when it is reversed. Waveforms after the polarity-reversing cochlear microphonic are typically absent or significantly aberrant^26,27^.

DPOAE testing was performed using a handheld otoacoustic emission instrument (Titan, Interacoustics A/S, Middelfart, DK, Denmark). Two stimulating pure tones, F1 and F2, were presented with L1 = L2 = 70 dB, and F2/F1 ratio of 1.22. The F2 frequencies were stepped through the range from 500 to 8000 Hz. DPOAEs were considered present if the signal-to-noise ratio was at least 3 dB. Passing scores had to be obtained for at least 4 of the 8 observed frequencies.

Acoustic immittance was carried out using a tympanometer (Tympstar, Grason-Stadler, Eden Prairie, MN, USA). The exclusion criteria for acoustic immittance were perforations, external ear canal atresia, purulent otorrhea and complete occlusion due to earwax. Tests with a 1000 Hz probe tone were carried out for infants up to 9 months of age, and tests with a 226 Hz probe tone were carried out for infants older than 9 months of age^27–29^. The probe tone presented a 75-dB SPL at 226 Hz or 1000 Hz. The velocity was 50 daPa/second, and the pressure was from + 200 to − 400 daPa. In relation to tympanometry with a 1000 Hz probe tone in infants, the single-peaked and double-peaked tympanograms were indicated to have normal middle ear function, and the flat-sloping and other unclassified tympanograms suggested abnormal middle ear function^29,30^. With regard to tympanometry with a 226 Hz probe tone, the type A curve (normal curve having a single peak of middle ear pressure between − 150 and + 100 daPa, admittance ranging from 0.2 to 1.6 cm^3^ and a ear canal volume of 0.2–1.8 mL) indicated normal middle ear function. and type B, C, As, and Ad curves implied abnormal middle ear function^31,32^.

### Definitions of HL

In this study, an audiometric air conduction threshold of c-ABR ≥ 30-dB nHL in any ear was considered HL^24,25^. The type of HL, which includes conductive HL (CHL), sensorineural HL (SNHL), mixed HL, and ANSD, was determined based on comprehensive results of c-ABR, DPOAE, acoustic immittance, temporal CT or inner ear MRI scan results. The degree of HL was graded by the hearing level of the worst ear in unilateral HL and by the hearing level of the best ear in bilateral HL. In this paper, the degree of HL was coded according to the air conduction thresholds of the c-ABR: normal (≤ 30 dB nHL), mild HL (31–50 dB nHL), moderate HL (51–70 dB nHL), severe HL (71–90 dB nHL) and profound HL (≥ 90 dB nHL)^24,33–35^.

### Data analysis

Statistical analysis was performed by using SPSS (SPSS Statistics version 26.0, IBM Corp., Armonk, NY, USA). Data were verified by another researcher. Counts and percentages were calculated to describe demographic characteristics, the distribution of hearing screening techniques, audiological outcomes and risk factors for HL. The means and standard deviations were derived for all parametric variables. The *χ*^2^ test was used to investigate between-group correspondence in screening results, audiological outcomes between groups screened by different hearing screening techniques and screening results between different sexes, as well as audiological outcomes between children with risk factors and children without risk factors. The two-sample *t* test was used to compare the average age at the first clinical visit between cases with and without risk factors. Risk factors for HL were analyzed using a binary logistic regression model, and the variables of age, sex, and eight risk factors for HL mentioned above were included in the initial model. Then, any variable with a *P* value < 0.1 in initial analysis was included in the multivariate conditional logistic regression model retained when the *P* value was < 0.05 using a forward elimination procedure. Different audiological outcomes based on UNHS outcomes were also analyzed by using a logistic regression model. Variables of different audiological outcomes (degree of HL including: mild, moderate, severe and profound) based on different UNHS outcomes (pass/referral) were initially included in an unadjusted analysis. Different UNHS outcomes (pass/referral) are independent variables. Different audiological outcomes (degree of HL including: mild, moderate, severe and profound) are dependent variables. Then, variables of age, sex and any variables with a *P* value < 0.1 in unadjusted analysis were included in the logistic regression model, with factors related to age and sex adjusted. Odds ratios (ORs) of HL and their 95% confidence intervals (CIs) were calculated for all variables in logistic regression analyses. A *P* value < 0.05 was considered to indicate statistically significance. Figures were generated using GraphPad Prism (GraphPad Prism version 9.3.1, GraphPad Software LLC., Boston, MA, USA) software version 9.3.1.

### Ethical approval

This study was approved by the Ethics Committee of Beijing Tongren Hospital, Capital Medical University.

## Results

### Demographic characteristics

**Figure 1:**
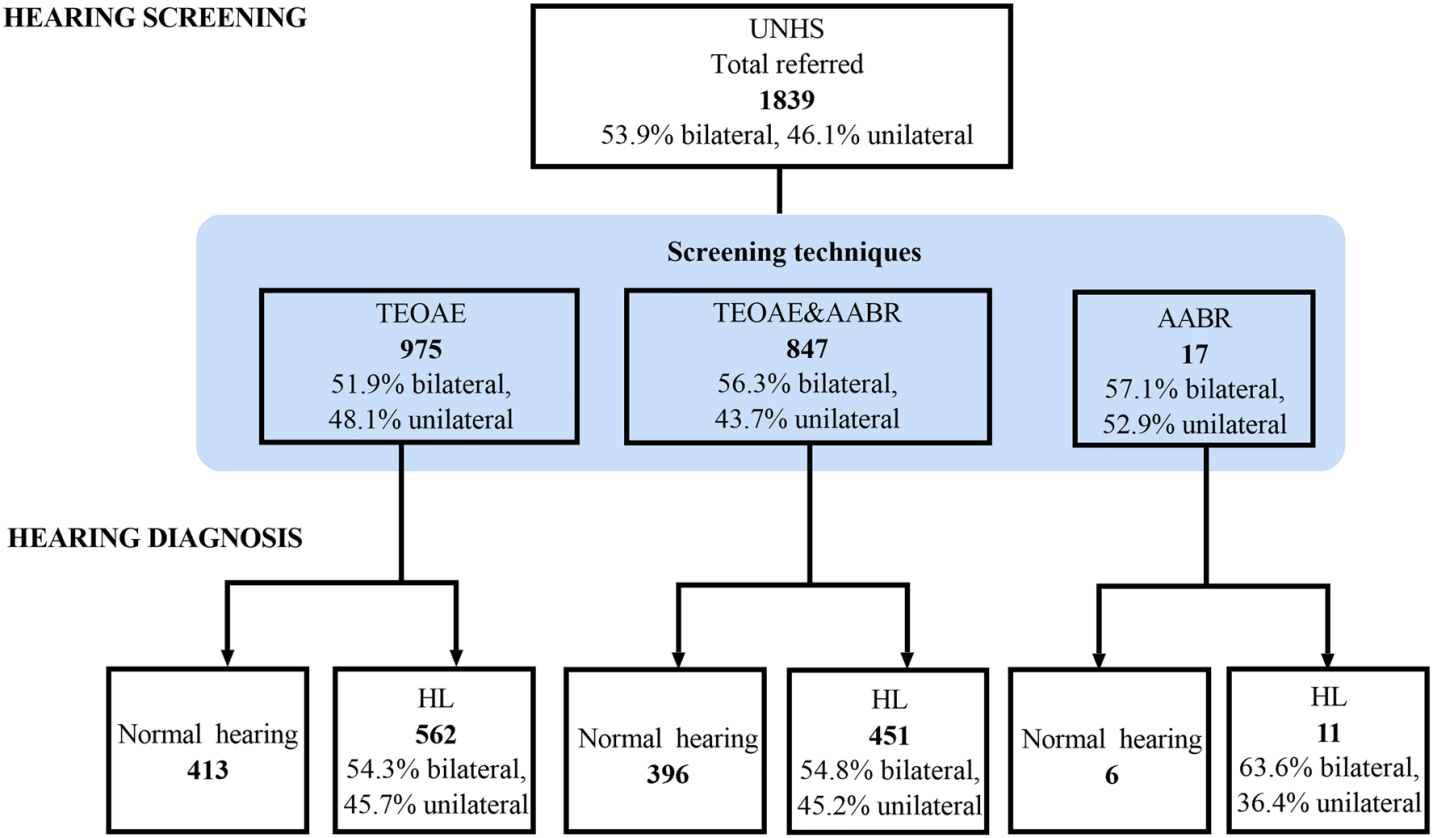
Flowchart of hearing screening and diagnosis for the study cohort.

**Figure 2:**
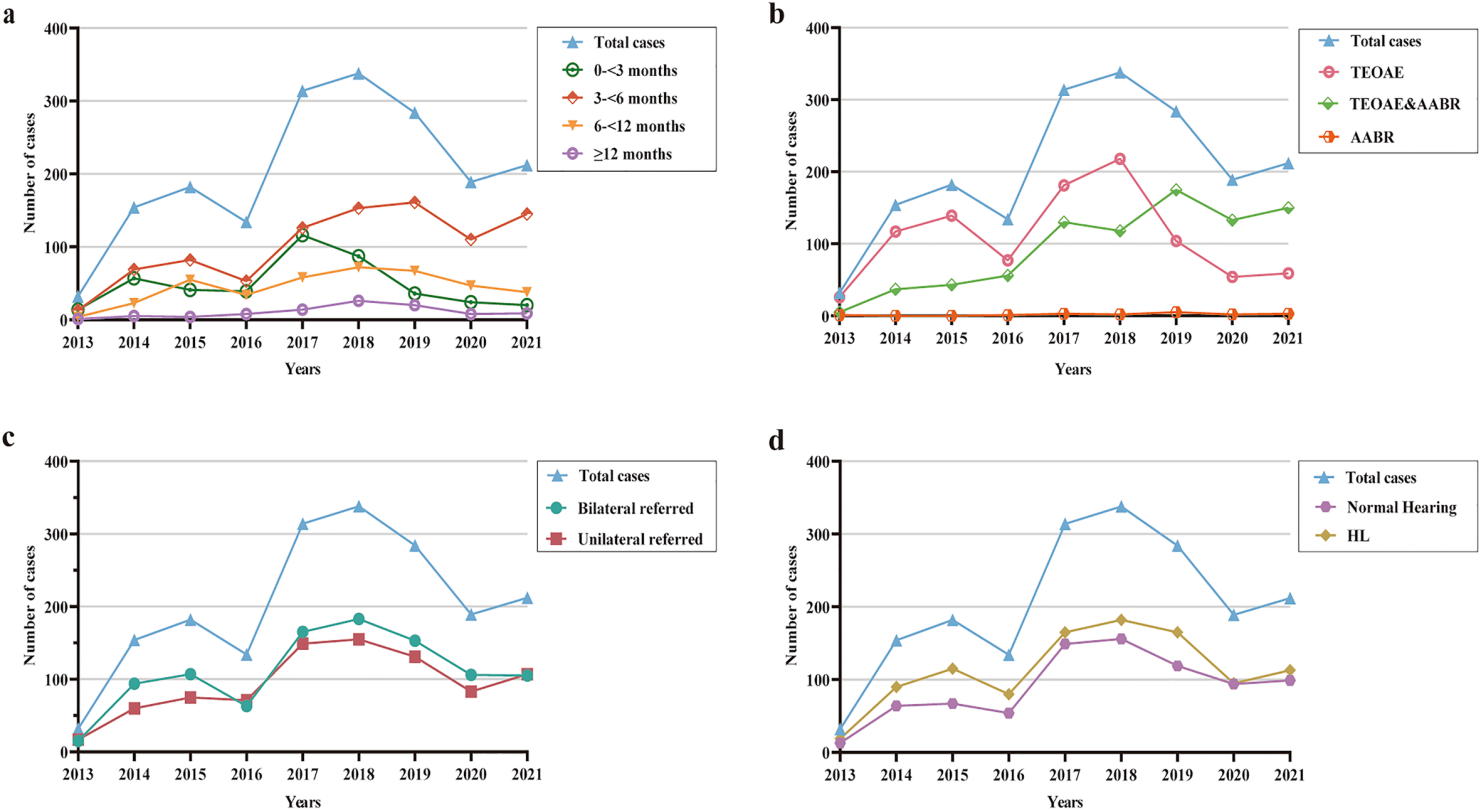
Number of cases over 9 years (n = 1839). (**a**) The distribution of different age groups at the first clinical visit; (**b**) the distribution of three hearing screening techniques; (**c**) the distribution of bilateral and unilateral referred laterals; (**d**) the distribution of audiological outcomes.

During the 9-year study period, 2790 cases were referred to our hospital from the UNHS in Beijing. Ultimately, 1839 cases were enrolled, and 951 cases were excluded due to incomplete or unmatched information. Figure 1 provides an overview of the study cohort. A total of 1086 (59.1%) cases were boys, and 753 (41.0%) were girls. The median age at first referral consultation after failing UNHS was 3.7 months, with 0.9 months (craniofacial anomalies) as the earliest and 40.1 months as the latest. The age group distribution at the first referral consultation over the 9 years is shown in Fig. 2a.

### Screening techniques and screening results

Regarding different hearing screening techniques, 975 cases (53.0%) were screened by only TEOAE testing, 847 cases (46.1%) were screened by a combination of TEOAE and AABR testing, and 17 cases (1.0%) were screened by only AABR testing. The number of cases screened by a combination of OAE and AABR testing showed an upward trend from 2013 to 2021 (Fig. 2b), increasing from 15.6% in 2012 to 70.8% in 2021.

A total of 991 cases (53.9%) were referred from UNHS for bilateral HL. A total of 848 (46.1%) were referred for unilateral HL, with 369 cases (20.1%) referred for right-sided HL and 479 (26%) for left-sided HL. The number of cases with referrals for bilateral and unilateral HL from UNHS over the 9 years is illustrated in Fig. 2c. No statistically significant difference between referrals for unilateral or bilateral failure among sexes was found (*P* = 0.43). There was no statistically significant difference between referrals for unilateral or bilateral failure among the three different screening techniques (*P* = 0.143).

### Audiological outcomes

In this study, 55.7% (1024/1839) of cases had confirmed HL (54.6% had bilateral HL, and 45.4% had unilateral HL). Consequently, normal hearing thresholds on both sides were obtained in 815 cases (44.3%, 55.3% with referral for unilateral failure and 44.7% with referral for bilateral failure). When the laterality of screening was compared with the diagnostic ABR, unilateral or bilateral HL found by screening was likewise confirmed in 808 cases (78.9% of cases with confirmed HL). Unilateral HL was detected in 135 cases (13.2%) with bilateral screening failure, whereas bilateral HL was found in 67 cases (6.5%) with unilateral screening failure. Fourteen cases (1.4%) with unilateral screening failure were diagnosed with unilateral HL in the other ear. Interestingly, almost all ears that passed UNHS but had an abnormal diagnostic ABR mainly showed mild or moderate HL (8.6%, 73/848), but 3 ears had severe HL, 5 ears had profound HL, and 2 ears had confirmed ANSD (1 with severe HL, 1 with profound HL). The number of cases with HL and normal hearing over the 9 years is illustrated in Fig. 2d. Of all 3678 ears, 43.0% presented abnormal ABR thresholds, corresponding to the degree of HL among abnormal ABR outcomes: mild (54.5%), moderate (22.4%), severe (6.9%) and profound (16.3%); CHL was present in 21.0% of cases, SNHL in 72.1%, mixed HL in 6.3% and ANSD in 0.5% (Table 1).

**Table 1:** Distribution of screening results and audiological outcomes according to different hearing screening techniques (N = 3678 ears).

Characteristics	No. of ears in screening techniques (%)			Total No. of ears (%)
	TEOAE	TEOAE and AABR	AABR	
Number of ears	n = 1950	n = 1694	n = 34	n = 3678
Screening results				
Passed	469 (24.1)	370 (21.8)	9 (26.5)	848 (23.1)
Referred	1481 (75.9)	1324 (78.2)	25 (73.5)	2830 (76.9)
Audiological outcomes				
Normal	1083 (55.5)	996 (58.8)	16 (47.1)	2095 (57.0)
HL	867 (44.5)	698 (41.2)	18 (52.9)	1583 (43.0)
Degree of HL				
Mild	487 (25.0)	366 (21.6)	9 (26.5)	862 (54.5)
Moderate	205 (10.5)	144 (8.5)	5 (14.7)	354 (22.4)
Severe	55 (2.8)	52 (3.1)	2 (5.9)	109 (6.9)
Profound	129 (6.2)	136 (8.0)	2 (5.9)	258 (16.3)
Type of HL				
CHL	174 (8.9)	158 (9.3)	1 (2.9)	333 (21.0)
SNHL	630 (32.3)	497 (29.3)	15 (44.1)	1142 (72.1)
Mixed HL	56 (2.9)	42 (2.5)	2 (5.9)	100 (6.3)
ANSD	7 (0.4)	1 (0.1)	0 (0)	8 (0.5)

### Distribution of screening results and audiological outcomes with different hearing screening techniques

The distribution of screening results and audiological outcomes according to different hearing screening techniques is shown in Table 1. There was no significant difference in screening results (pass/referral) between different screening techniques (*P* = 0.257). There was also no significant difference in the presence of HL (HL/normal hearing) between different screening techniques (*P* = 0.071).

With regard to the ORs of different HL degrees (mild, moderate, severe, and profound HL) based on UNHS outcomes (pass/referral), the unadjusted analysis and multivariable adjusted analysis are illustrated in Table 2. With normal hearing as reference, the odds ratios of mild [OR 7.700, 95% CI 5.840–10.152], moderate [OR 16.584, 95% CI 9.255–29.715], severe [OR 20.501, 95% CI 6.481–64.848], and profound [OR 29.414, 95% CI 12.081–71.616] HL increased when the ears’ screening results led to referral (*P* < 0.001) compared to when screening results passed.

**Table 2:** ORs of hearing diagnoses based on UNHS outcomes (pass/referral).

Variables	Unadjusted analysis		Multivariable adjusted analysis	
	OR (95% CI)	*P* value	OR (95% CI)	*P* value
	–	< 0.001 ^a^	–	–
Normal hearing (ref.) ^b^	[Reference]	–	[Reference]	–
Mild HL	5.107 (3.987–6.542)	< 0.001	7.700 (5.840–10.152)	< 0.001
Moderate HL	13.294 (7.523–23.492)	< 0.001	16.584 (9.255–29.715)	< 0.001
Severe HL	18.973 (6.043–59.569)	< 0.001	20.501 (6.481–64.848)	< 0.001
Profound HL	24.708 (10.239–59.625)	< 0.001	29.414 (12.081–71.616)	< 0.001

Predictor variables: UNHS outcomes (pass/referral).

*OR* odds ratio, *CI* confidence interval.

^a^*P* value for trend.

^b^Normal hearing for reference, normal hearing refers to audiometric air conduction threshold of c-ABR < 30 dB nHL.

### Risk factors associated with HL

A total of 113 cases had risk factors for HL; among them, 76 (67.3%) were confirmed to have HL. The age at first clinical visit in cases with risk factors (4.53 ± 2.81 months) was significantly younger than that in cases without risk factors (5.12 ± 3.75 months) (*P* = 0.035). A comparison of audiological outcomes between cases with risk factors and cases without risk factors is shown in Table 3. The most recurring risk factors were craniofacial anomalies, including auricle and canal anomalies (94.0% with confirmed HL), followed by a length of stay in the NICU longer than 5 days (34.1% with confirmed HL) and birth weight less than 1500 g (20.0% with confirmed HL).

**Table 3:** Comparison of audiological outcomes between cases with and without risk factors.

High-risk factors	n (%) of cases by audiological outcomes		Statistical analysis	
	HL	Normal hearing	*Χ*^*2*^	*P* value
Craniofacial anomalies^a^				
Yes	47 (94)	3 (6)	30.580	< 0.001
No	977 (54.6)	812 (45.4)		
NICU admission^b^				
Yes	15 (34.1)	29 (65.9)	8.516	0.004
No	1009 (56.2)	786 (43.8)		
Low birth weight^c^				
Yes	3 (20)	12 (80)	7.803	0.005
No	1021 (56)	803 (44)		
Mechanical ventilation^d^				
Yes	5 (50)	5 (50)	0.002	0.965
No	1019 (55.7)	810 (44.3)		
HL-associated syndromes^e^				
Yes	7 (87.5)	1 (12.5)	2.128	0.145
No	1017 (55.5)	814 (44.5)		
Hyperbilirubinemia^f^				
Yes	3 (60)	2 (40)	< 0.001	1.000
No	1021 (55.7)	813 (44.3)		
Bacterial meningitis				
Yes	1 (25)	3 (75)	0.537	0.464
No	1023 (56)	803 (44)		
In utero infection^g^				
Yes	2 (100)	0 (0)	0.303	0.582
No	1022 (55.6)	815 (44.4)		

*NICU* neonatal intensive care unit, *HL* hearing loss.

^a^Including auricle and canal anomalies.

^b^Length of stay in the NICU longer than 5 days.

^c^Birth weight less than 1500 g.

^d^Mechanical ventilation time over 48 h/respiratory distress episode.

^e^HL-associated syndromes in clinical/family history.

^f^Hyperbilirubinemia that met the requirements for blood exchange.

^g^Including cytomegalovirus, toxoplasmosis, etc.

The unadjusted and multivariate analyses of risk factors associated with HL in this study, including variables of age, sex and different HL-related risk factors, are presented in Table 4. While a number of variables increased the odds of HL, only four variables were independent predictors: age, craniofacial anomalies including auricle and canal anomalies, length of stay in the NICU longer than 5 days and birth weight less than 1500 g (all variables with a *P* < 0.1 were listed as significant variables in the unadjusted analysis). To gain more specific results, a logistic regression model adjusted for age, craniofacial anomalies including auricle and canal anomalies, length of stay in the NICU longer than 5 days and birth weight less than 1500 g was conducted. Subsequently, age, craniofacial anomalies and low birth weight showed statistically significant differences associated with the presence of HL (*P* < 0.05).

**Table 4:** Unadjusted and multivariable adjusted analyses of risk factors associated with HL.

Variables	Unadjusted analysis		Multivariable adjusted analysis	
	OR (95% CI)	*P* value	OR (95% CI)	*P* value
Age	1.063 (1.033–1.094)	< 0.001	1.070 (1.040–1.102)	< 0.001
Sex	1.044 (0.866–1.259)	0.653	**–**	–
Craniofacial anomalies ^a^	13.021 (4.038–41.988)	< 0.001	0.068 (0.021–0.221)	< 0.001
NICU ^b^	0.403 (0.215–0.757)	0.005	1.674 (0.824–3.400)	0.154
Low birth weight ^c^	0.197 (0.155–0.699)	0.012	4.537 (1.013–20.328)	0.048
Mechanical ventilation ^d^	0.795 (0.229–2.755)	0.717	**–**	–
HL-associated syndromes ^e^	5.603 (0.688–45.630)	0.107	–	–
Hyperbilirubinemia ^f^	1.194 (0.199–7.165)	0.846	–	–
Bacterial meningitis	0.265 (0.267–2.548)	0.25	–	–
In utero infection ^g^	> 999.99 (0.00– > 999.99)	0.999	–	–

*NICU* neonatal intensive care unit, *HL* hearing loss.

^a^Including auricle and canal anomalies.

^b^Length of stay in the NICU longer than 5 days.

^c^Birth weight less than 1500 g.

^d^Mechanical ventilation time over 48 h/respiratory distress episode.

^e^HL-associated syndromes in clinical/family history.

^f^Hyperbilirubinemia that met the requirements for blood exchange.

^g^Including cytomegalovirus, toxoplasmosis, etc.

## Discussion

Hearing screening, diagnosis and treatment are considered important and standard components of early interventions for HL among children, as well as ear and hearing care during the whole lifespan. Our study had one of the largest cohorts and provided an overview of UNHS and children’s HL characteristics during a 9-year period starting in 2013 in Beijing, China. In this study, 1839 newborns were referred after failing UNHS, and 55.7% of newborns were confirmed to have HL. According to other reports, HL is usually confirmed in 44–78% of newborns after failing UNHS^36–39^.

We found that the number of cases who had their first clinical visit between 3 and 6 months of age showed an upward trend over the 9 years; however, the number of cases who had their first clinical visit before the age of 3 months fluctuated, which might be related to the fluctuation in the yearly distribution of total cases. According to the Joint Committee on Infant Hearing (JCIH) Statement (2019) as well as China’s technical specifications (2010), all infants with HL should be identified as early as possible, and appropriate interventions should be initiated no later than at 3–6 months of age (screening completed by 1 month, audiologic diagnosis by 3 months, and enrollment in early intervention by 6 months). Our study indicated that an earlier diagnosis should be reinforced during the implementation of UNHS and children’s ear and hearing care in Beijing^11,27^. The number of cases screened by a combination of TEOAE and AABR testing showed an upward trend from 2013 to 2021. Until 2021, 70.8% of newborns were screened by a combination of TEOAE and AABR testing. China’s technical specifications (2010) recommend OAE or AABR testing in initial screening and rescreening for infants and AABR testing for NICU newborns. Wen et al. assessed the current status of UNHS at 26 institutions in China and reported that 73.08% of these organizations used a combination of TEOAE and AABR testing for screening^13^. Combining TEOAE and AABR testing is becoming a mainstream screening technique in the UNHS program in Beijing, China.

Van et al. reported that the use of TEOAE testing as a screening tool is likely to result in a higher failure rate in the immediate post birth period compared with AABR testing^40^. In our study, TEOAE testing resulted in a higher failure rate than AABR screening, but there was no statistically significant difference, which might be related to the sample size and screening period of children. It was also reported that OAE testing failure might be influenced by ear canal obstruction or middle ear effusion^41–44^. In our study, 8.9% of children screened by TEOAE testing and 9.3% screened by a combination of TEOAE and AABR testing had confirmed CHL, which was higher than the 2.9% of children screened by AABR testing, which might indicate that TEOAE testing can detect more defects in the ear canal and middle ear.

We also found that the odds ratio of mild, moderate, severe, and profound HL increased with UNHS referral outcomes than with UNHS passing outcomes, which showed that UNHS was an effective way to detect different degrees of HL, especially a more severe degree of HL.

The vast majority of children’s ears that passed screening and subsequent diagnostic ABR testing confirmed normal hearing, 8.6% had confirmed mild or moderate HL, and 0.9% had confirmed severe or profound HL. Interestingly, 2 ears had confirmed severe and profound ANSD, and they were screened by OAE testing only. TEOAE testing is a convenient method to assess the function of the peripheral auditory system, while AABR testing can detect defects in the whole auditory pathway, which compensates for the disadvantage that TEOAE testing cannot identify retro-cochlear hearing impairment^41–44^. Consistent with this, our study found that screening with TEOAE testing will result in missing children with ANSD, which explains why some children with ANSD passed UNHS at first and came to the clinic for further diagnosis late in their childhood, which should be given more importance during children’s ear and hearing care. In addition, either OAE or AABR screening techniques can miss cases with minimal-mild-moderate HL, which is consistent with the false-negative screening diagnosis outcomes above in our study^44,45^. Although UNHS cannot identify all infants with HL, the importance of acknowledging and estimating the occurrence of false-negative outcomes must continue to receive increasing attention in children’s ear and hearing care.

A low incidence of ANSD (0.50%) among all confirmed HL was revealed in the current study, which was lower than rates (1.81–5.42%) quoted in the literature^36,38,46^. This might be because the proportion of infants with risk factors for ANSD, including NICU stay, low birth weight, hyperbilirubinemia, and the use of mechanical ventilation, in the current study cohort was small^47–49^.

Depending on the risk factors, there is significant variation between published studies ragarding the most prevalent risk factors. A large cohort study by Zhou et al. on risk factors for HL among 7287 neonates in China showed that craniofacial malformations, NICU admission history and other risk factors were closely related to HL, and the top two recurring risk factors in this study were consistent with the above^46^. In another study, hyperbilirubinemia was presented as the most frequently associated risk factor; however, in our findings, the prevalence of hyperbilirubinemia was low among all risk factors found^50^. In addition, according to our statistical analysis, age, craniofacial malformations and low birth weight were associated with the presence of HL. These associations may indicate that more attention should be given in tertiary referral centers as well as in obstetrics departments and child health care departments in the early stages to allow for HL cases with risk factors to be identified when a child is still in the womb.

## Limitations

This study had the following limitations. First, the data of this study were extracted from a single center in Beijing, and the results only reflected the status of newborn hearing screening and diagnoses in Beijing Tongren Hospital, Capital Medical University, from 2012 to 2021. The status of newborn hearing screening in China should be derived from a comprehensive analysis of data from more regions. Second, the associations between risk factors and the presence of HL we observed may have been due to shared risk factors for the same case, which might be causal or may be spurious.

## Conclusion

In this paper, we presented our experience in the UNHS program for neonatal HL, specifically in relation to the comparison of screening and diagnosis over 9 years. We demonstrated that, despite having some limitations in external validity, there is valuable information to be gained on the advantages of carrying out UNHS, which is an important tool for detecting different degrees of HL among children in early stages. In addition, attention should be given to early diagnosis, intervention and follow-up for children with risk factors for HL, such as NICU admission history, craniofacial malformations and low birth weight.

## Data Availability

All data generated or analyzed during this study are included in this article, including the figures and the tables.
